# Novel *Saccharomyces uvarum* x *Saccharomyces kudriavzevii* synthetic hybrid with enhanced 2-phenylethanol production

**DOI:** 10.1186/s12934-024-02473-3

**Published:** 2024-07-19

**Authors:** Karolina Drężek, Zsuzsa Antunovics, Agnieszka Karolina Grabiec

**Affiliations:** 1 Department of Drug and Cosmetics Biotechnology, Faculty of Chemistry, Warsaw University of Technology, Warsaw, Poland; 2 Department of Genetics and Applied Microbiology, University of Debrecen, Debrecen, Hungary

**Keywords:** Hybrids, *Saccharomyces*, 2-phenylethanol, Ehrlich pathway

## Abstract

**Background:**

Over the last two decades, hybridization has been a powerful tool used to construct superior yeast for brewing and winemaking. Novel hybrids were primarily constructed using at least one *Saccharomyces cerevisiae* parent. However, little is known about hybrids used for other purposes, such as targeted flavor production, for example, 2-phenylethanol (2-PE). 2-PE, an aromatic compound widely utilised in the food, cosmetic, and pharmaceutical industries, presents challenges in biotechnological production due to its toxic nature. Consequently, to enhance productivity and tolerance to 2-PE, various strategies such as mutagenesis and genetic engineering are extensively explored to improved yeast strains. While biotechnological efforts have predominantly focused on *S. cerevisiae* for 2-PE production, other *Saccharomyces* species and their hybrids remain insufficiently described.

**Results:**

To address this gap, in this study, we analysed a new interspecies yeast hybrid, II/6, derived from *S. uvarum* and *S. kudriavzevii* parents, in terms of 2-PE bioconversion and resistance to its high concentration, comparing it with the parental strains. Two known media for 2-PE biotransformation and three different temperatures were used during this study to determine optimal conditions. In 72 h batch cultures, the II/6 hybrid achieved a maximum of 2.36 ± 0.03 g/L 2-PE, which was 2–20 times higher than the productivity of the parental strains. Our interest lay not only in determining whether the hybrid improved in productivity but also in assessing whether its susceptibility to high 2-PE titers was also mitigated. The results showed that the hybrid exhibited significantly greater resistance to the toxic product than the original strains.

**Conclusions:**

The conducted experiments have confirmed that hybridization is a promising method for modifying yeast strains. As a result, both 2-PE production yield and tolerance to its inhibitory effects can be increased. Furthermore, this strategy allows for the acquisition of non-GMO strains, alleviating concerns related to additional legislative requirements or consumer acceptance issues for producers. The findings obtained have the potential to contribute to the development of practical solutions in the future.

**Supplementary Information:**

The online version contains supplementary material available at 10.1186/s12934-024-02473-3.

## Introduction

In recent decades, numerous strategies have emerged to tailor yeast strains for specific purposes. One straightforward approach entails delving into the largely unexplored natural biodiversity [1]. Despite having identified and characterized only a small fraction of the overall natural microflora, scientists aspire to artificially enhance these strains. While genetic engineering has been predominant since the 1980s, it now contends with classical methods that harness the potential of environmentally sourced strains. Recent reports suggest that a return to classical methods could prove advantageous, enabling the exploitation of the boundless potential inherent in strains sourced from the environment [2].

A traditional method for yeast modification involves hybridization, wherein cells from individuals with distinct genotypes merge to generate hybrid organisms, also known as crossbreeds. Through this process, advantageous gene combinations and desirable characteristics may manifest in the resulting hybrids.

A substantial amount of molecular and genetic data indicates that interspecific hybridization is commonly observed among natural strains of *Saccharomyces* sensu stricto, likely owing to their adaptation to severe conditions [3]**.** The current yeast taxonomy recognizes nine “natural” *Saccharomyces* species: *S. arboricolus* (*S. arboricola*), *S. cariocanus, S. cerevisiae, S. kudriavzevii, S. mikatae, S. paradoxus*, *S. jurei*, *S. eubayanus* and *S. uvarum* (*S. bayanus var. uvarum* ) [4]. Over the past decade extensive research has focused on members of *S. cerevisiae*, with over 5000 publications annually. Additionally, studies on hybrids, averaging around 150 per year, have primarily centered around crosses involving at least one *S. cerevisiae* parent [5–7].

Recent efforts have been dedicated to generating de novo yeast hybrids, tapping into their potential for biofuels, brewing, and winemaking production [8–10]. Phenotypic improvements have been primarily achieved through techniques such as protoplast fusion, spore-to-spore or spore-to-cell mating, or rare mating (extensively reviewed in [11]). Interspecies hybrids are not merely selected for their ability to combine beneficial traits from parental strains. The genomes of both parents often undergo chromosomal rearrangements, mutations, and gene loss/duplication events, which also influence the composition of formed protein complexes. Consequently, new and improved phenotypes can arise due to heterosis [12].

Despite the considerable effort invested in creating hybrids for the liquor or biofuel industry, there is limited data available on crosses developed for other purposes. Some reports have indicated an enhanced aroma profile in hybrids; however, these have been analysed primarily in the context of the wine or beer industry, specifically during alcoholic fermentation [13]. Therefore, it appears intriguing to explore the potential for crossbreeding yeast strains to create new yeast lineages with unique phenotypes for alternate purposes, such as targeted flavour production.

One of the most commonly utilised fragrances in the perfumery industry is 2-phenylethanol (2-PE), an aliphatic alcohol known for its pleasant floral-rose aroma. In addition to its distinctive scent, 2-PE serves as an antimicrobial preservative in cosmetic products. Furthermore, it finds application in laundry and home care products. The market size for 2-PE surpassed $255 million in 2021, with an estimated compound annual growth rate of 5.5% between 2022 and 2028 [14].

2-PE naturally occurs in plants or can be synthetically manufactured from benzene. Over the past two decades, numerous studies have focused on developing biotechnological methods for 2-PE production, utilizing yeast, genetically modified bacteria, or filamentous fungi [15–17]. Despite numerous studies, 2-PE biosynthesis via Ehrlich Pathway still remains the most efficient in *S. cerevisiae* strains [18, 19].

Interestingly, information regarding other species within the *Saccharomyces* genus is limited. We believe that filling this knowledge gap is crucial. Hence, our objective was to explore 2-PE production in selected species of *S. uvarum* and *S. kudriavzevii*. We aimed to determine their potential as viable candidates for 2-PE biosynthesis and to compare their ability to produce 2-PE with that of *S. cerevisiae* strains. Given the propensity of *Saccharomyces* species for spontaneous hybridization [3], we were also interested in determining whether hybrids formed from these species could exhibit improved phenotypes.

## Materials and methods

### Yeast strain and media composition

The yeasts *Saccharomyces uvarum* JRY9193 SSS111 (*MATalpha*^*Su*^* ade2*^*Su*^* ura3*^*Su*^* ho*^*Su*^, id number 10–1651) [20], *Saccharomyces kudriavzevii* FM1193 SSS411 (*MATa*^*Sk*^* trp1*^*Sk*^* ura3*^*Sk*^* ho*^*Sk*^, id number 10–1653) [20], and interspecific hybrid *S. uvarum x S. kudriavzevii* II/6 *(MATa*^*Sk*^*/MATalpha*^*Su*^* ho*^*Sk*^*/ho*^*Su*^* ADE2*^*Sk*^*/ade2*^*Su*^* trp1*^*Sk*^*/TRP1*^*Su*^* ura3*^*Sk*^*/ura3*^*Su*^*)* [21] were used in the study. Data on obtaining the II/6 hybrid can be found in [22].

The strains were maintained on YPDA medium (2% (w/v) glucose (Bioshop, Burlington, Canada), 2% (w/v) bacteriological peptone (Bioshop), 1% (w/v) yeast extract (Bioshop), and 2% (w/v) agar (Bioshop) at 4 °C. Cultures for 2-PE exogenous effect on yeast growth were carried out in YPD (YPDA without agar). 2-PE de novo synthesis was examined in D1 medium containing 0.13% (w/v) YNB without amino acids (Bioshop) and 2% (w/v) glucose (Bioshop). 2-PE biosynthesis via Ehrlich Pathway was evaluated on NEO [18, 23] and P8 [24] media, containing L-Phenylalanine—the precursor for the biosynthesis of 2-PE. Additionally, D1 and P8 media were supplemented with the relevant amino acids; adenine (Sigma Aldrich, St. Louis, MO, USA) and uracil (Sigma Aldrich) for *S. uvarum* 10–1651*,* tryptophane (Sigma Aldrich) and uracil for *S. kudriavzevii* 10–1653, or uracil for II/6 hybrid, respectively.

### Biochemical characteristics

To assess spore formation ability, strains were streaked onto acetate medium and incubated at 25 °C for 5 days. They were subsequently examined under light microscopy (Olympus CH-2, Tokyo, Japan). Next, the spores from 10 tetrads of each sample were dissected (Zeiss 2588 micromanipulator, Jena, Germany), and colony formation was observed after another 5 days.

Strains were checked for the growth on various carbon sources using solid media containing: 0.67% (w/v) yeast nitrogen base with ammonium sulphate (Bioshop), 2% (w/v) agar and, respectively, 2% (w/v) D-glucose (Bioshop), D-fructose (Bioshop), D-xylose (Carl Roth, Karlsruhe, Germany), D-galactose (Carl Roth), D-maltose (Bioshop), lactose (Carl Roth), sucrose (Bioshop), cellobiose (Fluka, Buchs, Switzerland), ethanol (POCH, Gliwice, Poland), or glycerol (Bioshop) as a single carbon source at 30 °C for 2–4 days.

In addition, fermentation ability was tested on YPL (YPD containing 2% (w/v) melibiose, maltose and galactose instead of glucose). Fermentation tests were performed in test tubes with Durham tubes. Results (CO_2_–bubble-formation) were read after 14 days of incubation at 25 °C.

Furthermore, the yeast growth on rich solid YPD medium was also tested at various temperatures, between 4, 10, 15, 20, 25, 30, 35, 37 and 42 °C.

### Effect of exogenous 2-PE on yeast growth

To evaluate the effect of 2-phenylethanol, overnight cultures of *S. uvarum* 10–1651, *S. kudriavzevii* 10–1653, and II/6 hybrid were diluted in triplicate in 50 mL YPD medium to an optical density at 600 nm (OD600) of 0.15 and grown at 30 °C with shaking at 240 rpm for 48 h (LAB Companion SI-600R, Ramsey, MN, USA). When the cultures reached an OD600 value of approx. 0.6–0.8, 2-PE (Merck, Darmstadt, Germany) was added to the cultures to reach final concentrations of 0.5–3.0 g/L. A culture without exogenous 2-PE addition was conducted as a control. In addition to OD600, microscopic observations were conducted after 24 h of incubation. The images of unstained cells were captured through a 100 × objective lens under bright field microscopy (Nikon Eclipse Ni microscope, Plan Apo VC objective (100x/1.40 oil, OFN25 DIC N2), Nikon, Tokyo, Japan).

### 2-Phenylethanol production in batch cultures

A sterile loop full of biomass from a single colony was used to inoculate the YPD medium in a 100 mL Erlenmeyer flask and grown overnight at 30 °C. The overnight culture was then diluted in 50 mL of D1 medium (for evaluating de novo synthesis) or P8 supplemented with amino acids, and NEO media (for 2-PE production via Ehrlich Pathway) in a 100 mL Erlenmeyer flask to an OD600 of ~ 0.15. Batch cultures were incubated at selected temperatures (25 °C, 30 °C, and 35 °C) with shaking at 240 rpm (LAB Companion SI-600R, Ramsey, MN, USA) for 72 h in triplicate. 1 mL samples were collected at indicated time points to determine OD600, sugars, and 2-PE concentration.

### Analytical methods

2-PE and L-phe concentration was determined using a high-performance liquid chromatography, HPLC (SYKAM chromatograph, Sykam GmbH, Eresing, Germany), with a DAD detector and a Bionacom Velocity C18-2 column (250 × 4.6 mm, 5 μm). An isocratic method comprising 70:30 2% formic acid/acetonitrile with 2% formic acid was used at a flow rate of 1 mL/min. DAD detector was set at a wavelength of 256 nm. Glucose, sucrose and ethanol content was determined by HPLC coupled with an RI detector and a SETREX IEX H + column (300 × 8 mm column, Polymer IEX H form, 8 μm) under thermostatic control at 35 °C. The RI detector was also set at 35 °C to avoid fluctuations in detector responses. Samples were eluted isocratically using 9 mM H_2_SO_4_ as the mobile phase at a flow rate of 1 mL/min. The Clarity software was used to construct standard curves (glucose 1–100 g/L, sucrose 1–100 g/L, ethanol 1–60 g/L, L-Phe 1–4 g/L, 2-PE 1–3 g/L) and to integrate the obtained data.

The cell density in liquid medium samples was monitored by measuring turbidity at 600 nm (OD600) using a VIS-7220G spectrophotometer (Beijing Rayleigh Analytical Instrument Corporation Co., Ltd., Beijing, China).

### Calculations

$$Specific \,growth\, rate \left( \mu \right)\, \mu = \frac{1}{{OD_{600} }}\frac{{dOD_{600} }}{dt} \left( \frac{1}{h} \right)$$

$$Space~time~yield~\left( {P_{{2PE}} } \right)~\,P_{{2PE}} = ~\frac{{\Delta C_{{2PE}} }}{{\Delta t}}~\left( {\frac{{\frac{{mg}}{L}}}{h}} \right)$$

$$\Delta C_{2PE} = C_{2PE} - C_{2PE.0h}$$

where OD_600_ is a turbidity at λ = 600 nm, ∆C_2PE_ is the ratio of the achieved concentration of the product (2-PE), C_2PE_ is 2-PE titer (mg/L) estimated at specific time points (24 h or 72 h), and C_2PE.0 h_ is 2-PE titer (mg/L) at the beginning of the cultures (0 h), which equals 0.

### Statistical analysis

Data are expressed as the mean ± SD (n = 6) obtained from three independent experiments measured in duplicate. Statistical comparisons were performed between groups using Student’s unpaired t-tests; p _value_ = 0.05 was the criterion for statistical significance.

## Results and discussion

### Metabolic characterisation of chosen *Saccharomyces* yeasts

The combination of gene pools from parental strains within *Saccharomyces* species, achieved through natural cell mating and subsequent meiotic and mitotic segregation, produces a novel strain with significant potential across various industries. However, hybridization can result in both advantageous and disadvantageous phenotypes [25]. In previous studies, an interspecific hybrid (*S. uvarum* x *S. kudriavzevii* II/6) was constructed using the cross-replica technique [21, 22]. In this study, we aimed to assess the biotechnological potential of the obtained hybrid and compare it with its parental strains. We focused on evaluating targeted 2-PE production. Before delving into this, we analysed the physiological traits of our strains. To determine the taxonomic affiliations, we tested the type strains *S. uvarum* 10–1651, *S. kudriavzevii* 10–1653, and the interspecific hybrid *S. uvarum x S. kudriavzevii* II/6 for sporulation; growth at various temperatures; assimilation of single carbon sources such as D-glucose, D-fructose, sucrose, xylose, cellobiose, galactose, maltose, lactose, glycerol, and ethanol; fermentation of melibiose, maltose, and galactose; and extracellular enzymatic activity (Table 1).

**Table 1:** Results of physiological tests of *S. uvarum* 10–1651, *S. kudriavzevii* 10–1653, and II/6 hybrid

Strain	*S. uvarum* 10–1651	*S. kudriavzevii* 10–1653	II/6 hybrid
Sporulation	−	−	+
Growth	10–35 °C	10–30 °C	10–35 °C
Assimilation			
d-glucose	+	+	+
d-fructose	+	+	+
Sucrose	+	+	+
Xylose	−	−	−
Cellobiose	−	−	−
Galactose	+	−	+
Maltose	+	+	+
Lactose	−	−	−
Glycerol	+	−	+
Ethanol	−	−	−
Fermentation			
Melibiose	+	−	+
Maltose	+	+	+
Galactose	+	+	+
Extracellular hydrolases secretion			
Lipolytic	+	−	+
Proteolytic	−	−	−
Amylolytic	−	−	−

The parental strains did not produced spores. The interspecific hybrid strain II/6 did produce spores, but none of them were viable. Strains were able to grow between 10 °C and 35 °C, except *S. kudriavzevii* 10–1653, which showed good growth up to 30 °C. All of tested strains were glucose, fructose, sucrose, and maltose positive. None of the strains demonstrated the ability to assimilate xylose, lactose, cellobiose, or ethanol. Additionally, *S. uvarum* 10–1651, and II/6 hybrid displayed the capacity to metabolise galactose and glycerol. Furthermore, fermentation of melibiose, galactose, and maltose was observed for all variants tested, with exception of *S. kudriavzevii* 10–1653, which did not exhibit fermentation of melibiose. When analysing the ability to produce extracellular hydrolases, only weak lipolytic activity was observed for *S. uvarum* 10–1651 and II/6 hybrid (Table 1).

Considering all the results, it becomes apparent that hybrid II/6 has inherited all the traits of the parental strains without manifesting any additional characteristics.

### 2-PE synthesis through primary metabolism

Yeasts can generate 2-PE either through de novo synthesis from glucose via the shikimate pathway or by catabolism of exogenous L-phenylalanine (L-phe) through the Ehrlich pathway. The Ehrlich pathway predominates over de novo synthesis when L-phe is the sole nitrogen source in the medium. In contrast, de novo synthesis typically dominates at low amino acid concentrations. The concentration of 2-PE produced through normal metabolism is rather low [19]. Therefore, de novo synthesis of 2-PE is not a viable route for economical bioprocesses.

While de novo biosynthesis is not particularly relevant when aiming to produce 2-PE on an industrial scale, it was interesting to investigate whether the new hybrid exhibited any differences compared to the parental strains. To achieve this, we conducted 72-h cultures in D1 medium, during which we monitored yeast growth by measuring optical density at indicated time points. Moreover, after 24 h, 48 h and 72 h samples to determine 2-PE concentration in culture broth were collected (Table 2).

**Fig. 1:**
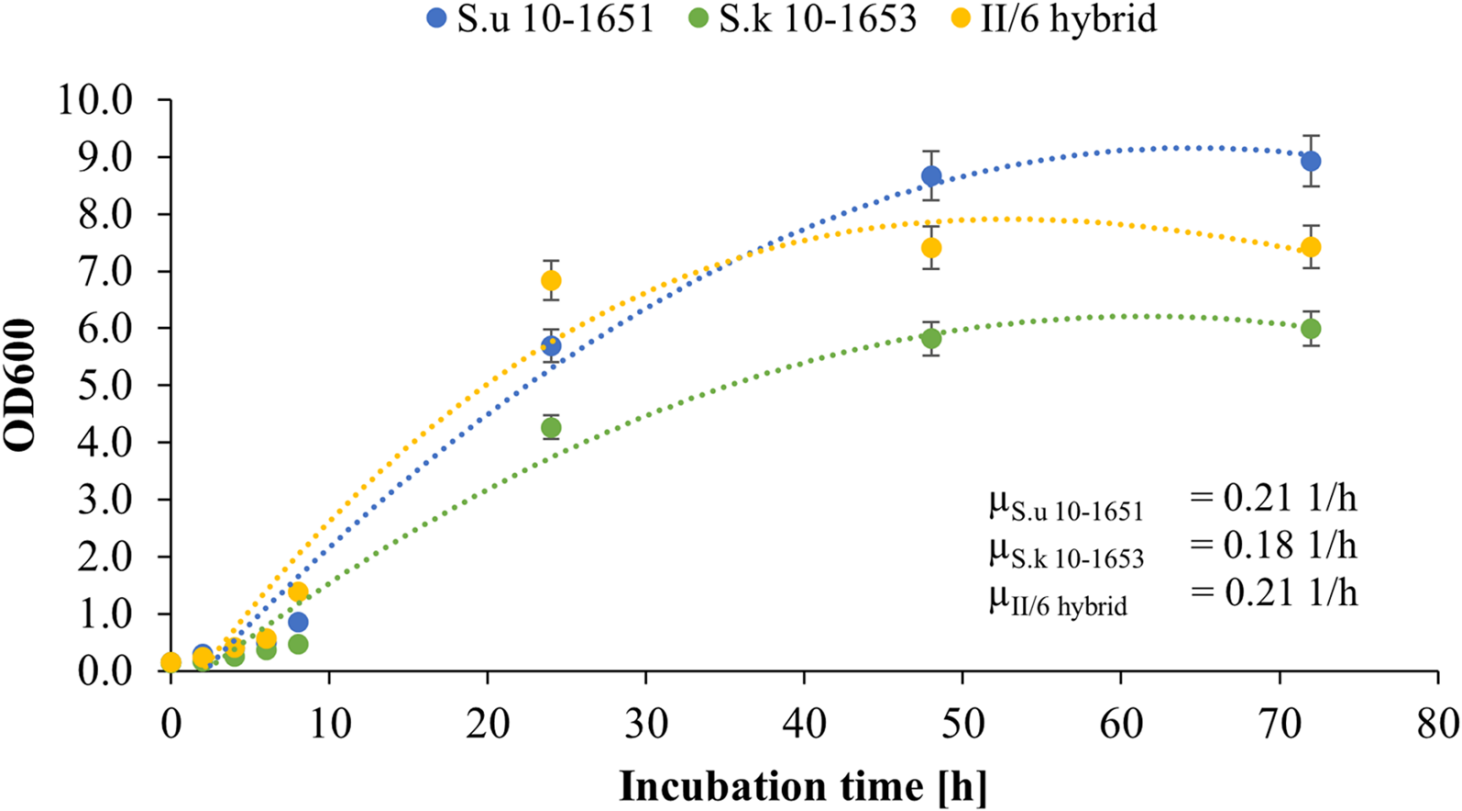
Growth monitoring in a 72-h batch cultures of *S. uvarum* 10–1651, *S. kudriavzevii* 10–1653, and II/6 hybrid in D1 medium at 30 °C. Cultures were performed in triplicate and data are presented as the mean ± SD

As illustrated in Fig. 1 parental strain S.u and II/6 hybrids displayed similar growth rates (μ) of 0.21 1/h. Yet, at the end of the cultures, 17% lower OD600 value was observed for II/6 hybrid (7.423 ± 0.091 *vs* 8.929 ± 0.040). As for S.k strain, although the estimated specific growth rate was only 14% lower (0.18 1/h), the final OD600 value for this strain was reduced by 20–33% (5.997 ± 0.089).

Analysing the results obtained (Table 2), there was no 2-PE production after 24 h of culture. For the S.u and II/6 hybrid strains, the maximum 2-PE titre was determined after 48 h, with concentrations of 64.5 mg/L and 34 mg/L, respectively. After 72 h, we noted a decrease in 2-PE concentration. In contrast, for S.k, 2-PE production progressed steadily over the 72 h period. At the end of the cultures, 55 mg/L of 2-PE was determined.

**Table 2:** 2-PE production by *S. uvarum* 10–1651, *S. kudriavzevii* 10–1653, and II/6 hybrid after 24 h, 48 h, and 72 h of incubation in shaking flasks in YPD medium at 30 °C

Strain	2-PE [mg/L]		
	24 h	48 h	72 h
*S. uvarum* 10–1651	nd	64.5 ± 5.5	40.0 ± 3.0
*S. kudriavzevii* 10–1653	nd	50.5 ± 1.5	55.0 ± 5.0
II/6	nd	34.0 ± 5.7	11.5 ± 2.1

nd–not detected

### Evaluation of 2-PE coming from catabolism of exogenous L-phenylalanine in batch culture

**Fig. 2:**
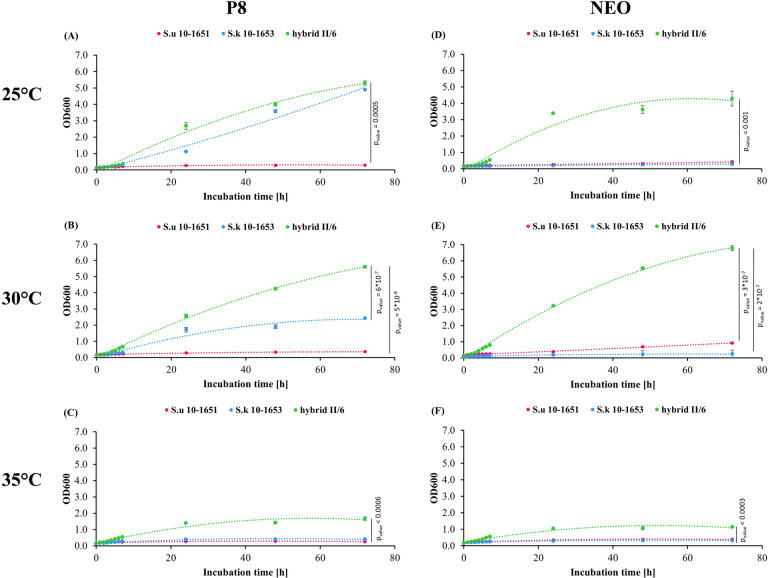
Growth monitoring in a 72-h batch cultures of *S. uvarum* 10–1651, *S. kudriavzevii* 10–1653, and II/6 hybrid in P8 (**A**–**C**) and NEO (**D**–**F**) media at 25 °C, 30 °C, and 35 °C. For each strain, three 72-h batch cultures were conducted. Analyses were performed in triplicate and data are presented as the mean ± SD

We conducted a series of 72 h batch cultures to evaluate 2-PE production coming from the catabolism of exogenous L-phenylalanine in two media specific for L-Phe biotransformation, differing in carbon and nitrogen sources, their concentrations, and L-phe amount. While NEO contains primarily glucose, yeast extract and L-phe [23], medium P8 is composed of glucose and sucrose, YNB without amino acids and ammonium sulfate and L-Phe [24], so as to ensure the most favourable conditions for 2-PE production along the Ehrlich route. Three different temperatures were tested: 25 °C, 30 °C and 35 °C. During cultures, OD600, 2-PE, glucose, sucrose, ethanol were monitored at 24 h, 48 h and 72 h (Fig. 2, Table 3).

**Table 3:** Basic parameters of the 72-h batch cultures of *S. uvarum* 10–1651, *S. kudriavzevii* 10–1653, and II/6 hybrid for 2-PE bioproduction via Ehrlich pathway in shaking flasks in P8 and NEO media at 25 °C, 30 °C, and 35 °C

	Final OD600	Glucose [g/L]	Sucrose [g/L]	Ethanol [g/L]	2-PE [g/L]	P_2-PE.max_^a^ [mg/L/h]	P_2-PE.total_^b^ [mg/L/h]
*P8*, *25 °C*							
*S*. *uvarum* 10–1651	0.30 ± 0.00	13.09 ± 0.82	–	1.77 ± 0.02	–	< 10	< 10
*S*. *kudriavzevii* 10–1653	4.89 ± 0.02	–	–	6 29 ± 0.19	0.85 ± 0.02	12.46 ± 0.08	11.77 ± 0.26
II/6	5.30 ± 0.13	–	–	6.13 ± 0.49	1.71 ± 0.03	40.14 ± 2.51	23.78 ± 0.46
*P8,* *30 °C*							
*S*. *uvarum* 10–1651	0.36 ± 0.02	8.35 ± 0.18	–	–	0.24 ± 0.01	< 10	< 10
*S*. *kudriavzevii* 10–1653	2.42 ± 0.05	–	–	2.43 ± 0.01	1.00 ± 0.01	28.35 ± 0.52	13.72 ± 0.03
II/6	5.61 ± 0.08	–	–	–	1.96 ± 0.08	37.75 ± 0.98	27.20 ± 1.07
*P8*, *35 °C*							
*S*. *uvarum* 10–1651	0.26 ± 0.01	17.35 ± 0.85	–	–	0.09 ± 0.00	< 10	< 10
*S*. *kudriavzevii* 10–1653	0.40 ± 0.04	11.73 ± 0.56	–	1.90 ± 0.13	0.15 ± 0.03	< 10	< 10
*II/6*	1.67 ± 0.11	5.26 ± 0.20	–	3.80 ± 0.01	0.54 ± 0.02	22.24 ± 0.76	< 10
*NEO*, *25 °C*							
*S. uvarum* 10–1651	0.42 ± 0.06	15.91 ± 1.15	x	1.86 ± 0.04	0.16 ± 0.00	< 10	< 10
*S*. *kudriavzevii* 10–1653	0.29 ± 0.05	18.54 ± 0.73	x	–	0.13 ± 0.01	< 10	< 10
*II/6*	4.28 ± 0.45	–	x	5.34 ± 0.18	1.41 ± 0.15	36.97 ± 1.31	19.64 ± 2.12
*NEO*, *30 °C*							
*S*. *uvarum* 10–1651	0.92 ± 0.02	14.49 ± 0.36	x	2.03 ± 0.09	0.37 ± 0.01	< 10	< 10
*S*. *kudriavzevii* 10–1653	0.24 ± 0.01	20.14 ± 0.15	x	1.07 ± 0.07	0.12 ± 0.01	< 10	< 10
*II/6*	6.78 ± 0.15	2.31 ± 0.12	x	1.29 ± 0.36	2.36 ± 0.03	40.94 ± 0.77	32.78 ± 0.37
*NEO*, *35 °C*							
*S*. *uvarum* 10–1651	0.39 ± 0.01	18.23 ± 0.71	x	–	0.16 ± 0.01	< 10	< 10
*S*. *kudriavzevii* 10–1653	0.32 ± 0.05	16.58 ± 0.00	x	1.54 ± 0.01	0.15 ± 0.04	< 10	< 10
*II/6*	1.14 ± 0.04	17.59 ± 0.21	x	2.99 ± 0.19	0.23 ± 0.00	< 10	< 10

^a^ – estimated after 24 h of incubation; ^b^ – estimated at the end of the culture; - –not present (= 0 g/L); x – absent in medium

The parental strains S.u and S.k predominantly exhibited limited growth under the tested conditions. In stark contrast, the hybrid yeast demonstrated growth rates up to fifteen times more robust across all variants. The optimal temperature for growth in both media tested was 30 °C, yielding final OD600 values of 5.61 ± 0.08 (P8) and 6.78 ± 0.15 (NEO). Growth at 25 °C resulted in final OD600 values 5–37% lower. The least favorable growth occurred at 35 °C. The calculated maximum specific growth rate was nearly identical in both tested media, registering at 0.23 ± 0.01 1/h (P8, 30 °C) and 0.24 ± 0.01 1/h (NEO, 30 °C).

Growth exhibited a clear correlation with sugar metabolism. Where growth was pronounced, nearly total glucose (or glucose and sucrose) consumption was observed. During the course of the cultivation we also monitored ethanol formation, as its high content may additionally inhibited 2-PE bioconversion [26]. Importantly, determined concentration did not exceed 0.5% in most cases.

The analysis of 2-PE production also revealed a noticeable correlation between growth and the final 2-PE titer. The highest concentrations of 2-PE, 2.36 ± 0.03 g/L and 1.96 ± 0.08 g/L, were achieved in a 72-h culture of the II/6 double hybrid at 30 °C in NEO and P8 medium, respectively. These values were approximately 2 (1.96 g/L vs 1.00 g/L, II/6 vs S.k) to 8.2 times higher (1.96 g/L vs 0.24 g/L) in medium P8 and between 6.4 (2.36 g/L vs 0.37 g/L) to almost 20 times higher (2.36 g/L vs 0.12 g/L) in medium NEO than the concentrations obtained from the culture of the parent strains.

The results obtained in cultures of parental strains (S.u and S.k) are comparable to or competitive with previously reported values. Tapia et al. [27] studied aroma production during wine fermentation and observed differences in 2-PE titers among various *Saccharomyces* species. They demonstrated that *S. uvarum* and *S. kudriavzevii* produce slightly higher concentrations of 2-PE from L-phe, with values not exceeding 0.9 g/L. This difference was attributed to varying activities of the biosynthetic enzymes involved. Similarly, Stribny et al. [28] reported distinctions between *S. kudriavzevii* and *S. uvarum* compared to *S. cerevisiae* in the production of higher alcohols and acetate esters using their amino acid precursors. Their studies also indicated higher 2-PE concentrations, although in this case, 2-PE titers remained below 270 mg/L. Nevertheless, compared to the results of other groups of 2-PE production by yeast screening among *Saccharomyces* species, values presented here are satisfactory [18, 29].

More importantly, our studies have demonstrated that hybridization can be a valuable approach for enhancing strains in targeted biotransformation of 2-PE. While the parental strains did not exceed 1 g/L in 2-PE bioconversion, the hybrid strain exhibited a significant improvement in production. In comparison to other strains obtained through genetic engineering or mutagenesis techniques, the results presented here are also promising [30–33].

Furthermore, the results presented here constitute the initial data on the analysis of the synthetic *S. uvarum* x *S. kudriavzevii* hybrid in terms of 2-PE production. Previously, we conducted an analysis of the *S. cerevisiae* x *S. cerevisiae* cross, which demonstrated enhanced 2-PE synthesis and improved resistance to its high concentration [34]. This phenotype has been reaffirmed in the current study. Hence, our findings can serve as a foundation for potential enhancements introduced into strains exhibiting high activity in the biotransformation of 2-PE.

### Effect of exogenous 2-PE on yeast growth

The II/6 hybrid strain exhibited enhanced production of 2-PE compared to the parental strains S.u and S.k. This enhancement can potentially be attributed to heightened physiological activity under the tested conditions. Furthermore, the II/6 strain displayed superior growth performance compared to the parental strains in media specifically designed for the bioconversion of L-Phe to 2-PE. This may account for the increased 2-PE production.

**Fig. 3:**
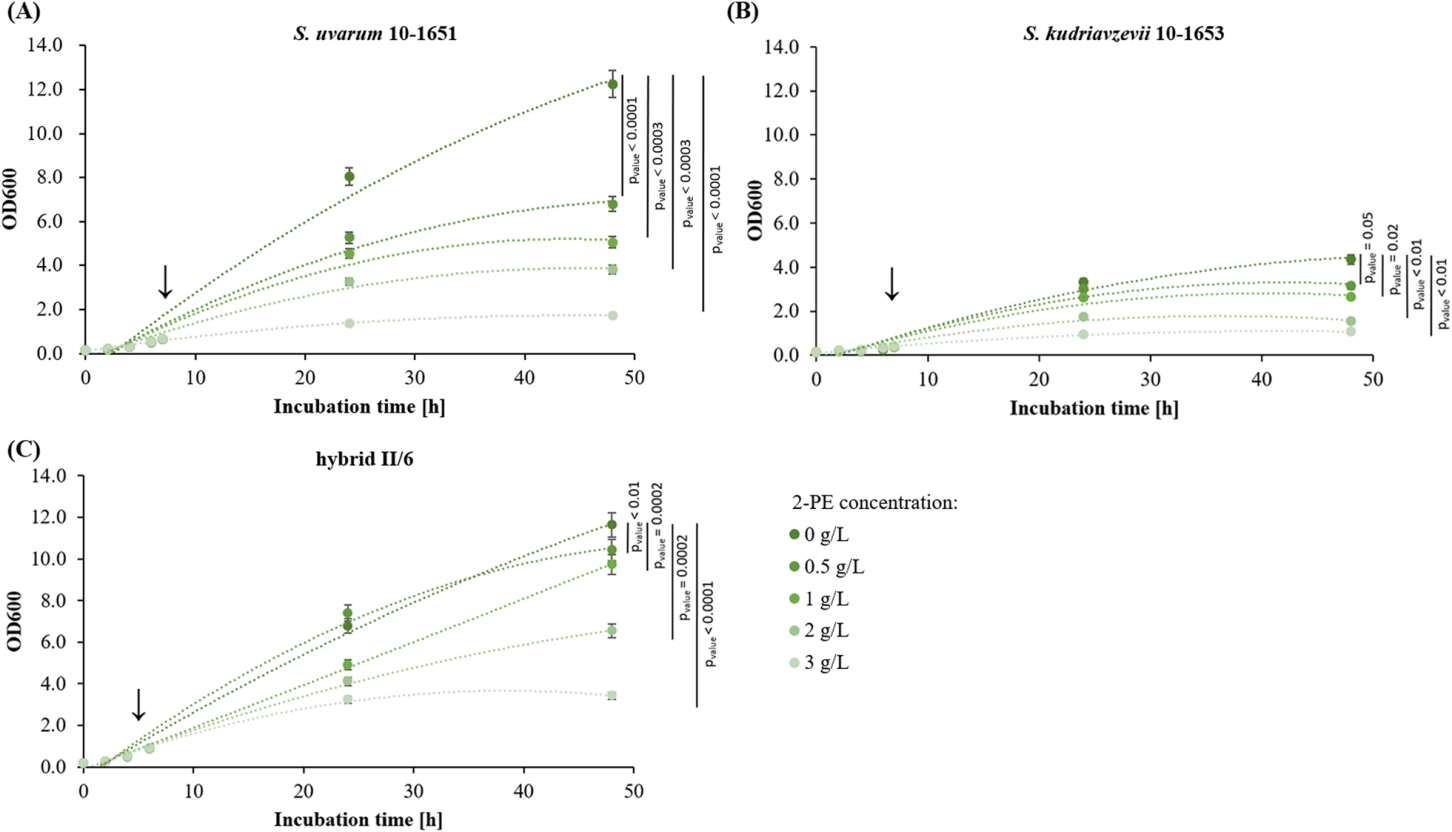
Effect of the presence of exogenous 2-PE on the growth of (**A**) *S. uvarum* 10–1651, (**B**) *S. kudriavzevii* 10–1653 and (**C**) II/6 hybrid strains. For each strain, five 48-h batch cultures in YPD were conducted. When the cultures reached OD600 values of 0.6–0.8 (indicated by an arrow), 2-PE was added to four cultures to final concentrations of 0.5, 1, 2, and 3 g/L, respectively. The fifth culture was not supplemented with 2-PE and served as a control. Analyses were performed in triplicate and data are presented as the mean ± SD

To investigate whether hybridization influenced the susceptibility of yeast cells to elevated 2-PE levels in the broth, we conducted 48 h batch cultures of II/6 and the parental strains S.u and S.k in YPD, supplemented with exogenous 2-PE to achieve final concentrations of 0.5, 1, 2, or 3 g/L. We monitored growth by measuring OD600 and observed cellular morphology through microscopic examinations (refer to Fig. 3 and supplementary Fig. S2).

Significant differences in growth were observed between the tested strains. The II/6 hybrid exhibited the highest resistance to the increased 2-PE levels, while *S. uvarum* 10–1651 showed the lowest resistance. In the presence of 1 g of 2-PE per liter in the culture medium, the growth of the II/6 strain decreased by 16% (p_value_ = 0.0002), while the growth of the parental strains decreased by 43%, p_value_ = 0.0003 (10–1651) and 39%, p_value_ = 0.0241 (10–1653), respectively. At 2 g of 2-PE per liter, there was a 69% (p_value_ = 0.0002) and 64% (p_value_ = 0.008) reduction in the final OD600 for S.u and S.k, respectively, with a 44% (p_value_ = 0.00003) decrease for the II/6 hybrid. At a 2-PE concentration of 3 g/L for all tested strains, growth was significantly inhibited by 86% (p_value_ = 0.00003), 75% (p_value_ = 0.006), and 71% (p_value_ = 0.0002).

In addition to monitoring yeast growth, we conducted microscopic observations to detect any changes in cellular morphology. At 2-PE concentrations ranging from 0.5 to 3 g/L, no significant differences were observed for all tested strains (please refer to Fig. S1).

The biological grounds for the substantial improvement in the resistance of the hybrid strain to 2-PE is compelling, motivating the continuation of research to elucidate the factors responsible for these changes. In a study conducted by Holyavkin et al. [35], the authors employed in vivo evolutionary engineering to adapt the yeast *S. cerevisiae* to 2-PE. Through genomic analyses, they identified potential point mutations in several genes, with the key mutation located in HOG1, encoding the Mitogen-Activated Kinase of the high-osmolarity signaling pathway. These findings were further supported by transcriptomic data. Consequently, exploring this aspect in future studies will be of our particular interest, inter alia, verification whether similar changes are behind the improved phenotype.

## Conclusions

In this current investigation, we have successfully characterized strains of *Saccharomyces uvarum* and *Saccharomyces kudriavzevii* as producers of 2-phenylethanol (2-PE). Additionally, for the first time, we have identified a newly constructed II/6 hybrid with an augmented 2-PE production capacity. Our findings reveal that this innovative hybrid not only surpasses the parental strains in 2-PE synthesis, exceeding their output by as much as 20 times, but also demonstrates superior resistance to high concentrations of 2-PE.

Moving forward, our next objective is to delve into the identification of the underlying biological factors contributing to the heightened biotechnological potency observed in this hybrid. The work presented herein not only showcases a beneficial use of hybridisation for yeast modification geared towards the production of naturally sourced 2-PE through yeast biotransformation but also underscores its biotechnological significance for synthesizing high value-added flavors. This study lays the foundation for further exploration into the intricate mechanisms behind yeast-mediated 2-PE production, thereby advancing our understanding and potential applications in the realm of flavor synthesis.

### Supplementary Information

Supplementary Material 1.

## Data Availability

The authors declare that the data supporting the findings of this study are available within the paper and its Supplementary Information files. Should any raw data files be needed in another format they are available from the corresponding author upon reasonable request.

## Abbreviations

2-PE: 2-Phenylethanol
L-phe: L-phenylalanine
OD600: Optical density at λ = 600 nm
